# Supplementary material for: Potentiation of Cisplatin Cytotoxicity in Resistant Ovarian Cancer SKOV3/Cisplatin Cells by Quercetin Pre-Treatment
Source: Int J Mol Sci. 2023 Jun 30;24(13):10960. doi: 10.3390/ijms241310960 (PMC10341453; doi:10.3390/ijms241310960)
Supplement: Supplementary file 1 [file ijms-24-10960-s001.zip › ijms-2432023-supplementary.pdf]

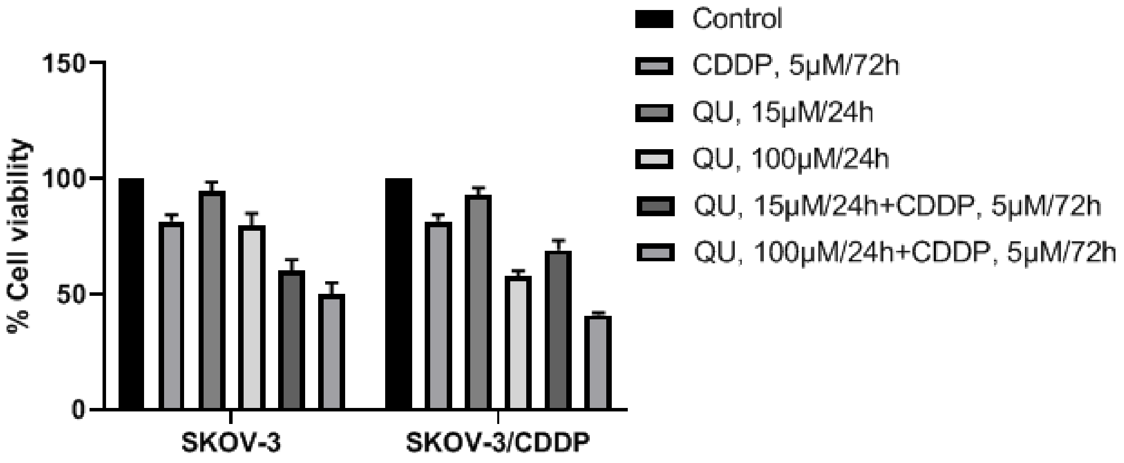

Figure S1. QU pre-treatment effect on the CDDP efficacy . The both types of cells were treated with low and high QU (15 or 100 μM) doses for 24 h followed by fresh medium for 72 h or /and CDDP (5 μM for SKOV-3 and 17 μM for SKOV-3/CDDP) for 72 h. Cell viability was assessed using MTT assay. All data are presented as mean ± SEM.

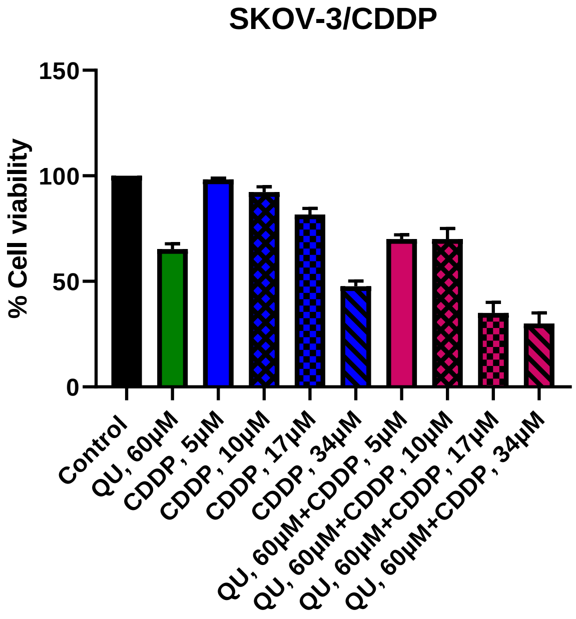

Figure S2. QU pre-treatment effect on the CDDP efficacy. The SKOV-3/CDDP cells were treated with QU (60 μM) for 48 h followed by fresh medium for 72 h or /and CDDP (5,10,17 and 34 μM) for 72 h. Cell viability was assessed using MTT assay.

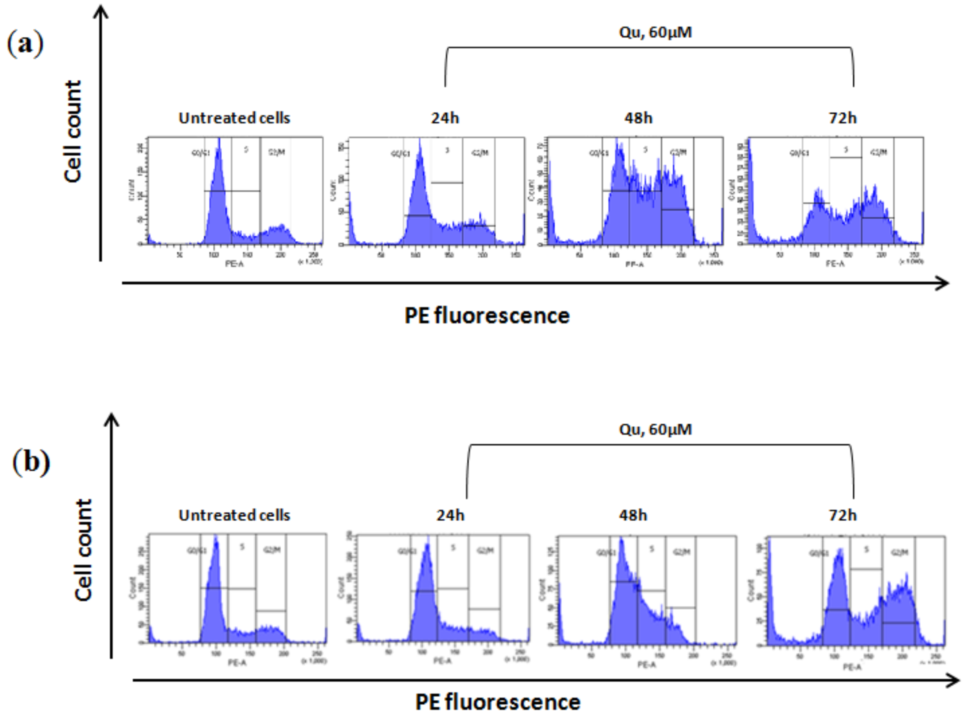

Figure S3. Representative cell cycle flow cytometry dot-plots of ovarian cancer (**a**)SKOV-3 and (**b**) SKOV-3/CDDP cells treated with (60 µM) QU for 24, 48 and 72 h.

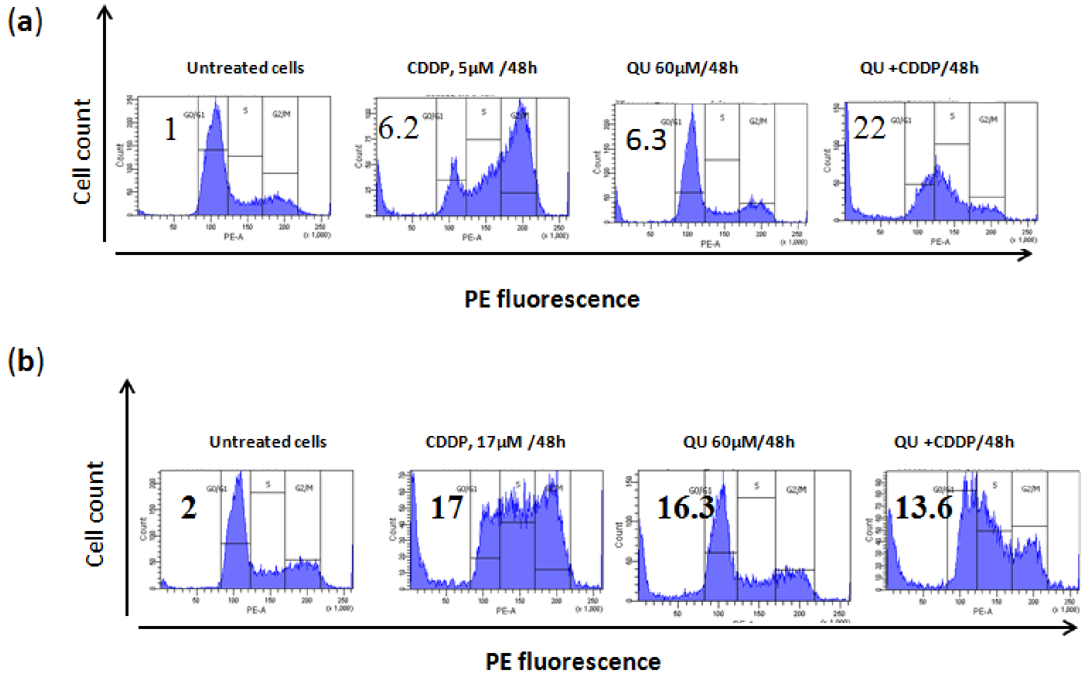

Figure S4. Representative cell cycle flow cytometry dot-plots of ovarian cancer (**a**) SKOV-3 and (**b**) SKOV-3/CDDP cells treated with (60 µM) QU or CDDP only and a combination QU and CDDP treatment for 48h.
